# Permafrost dynamics and the risk of anthrax transmission: a modelling study

**DOI:** 10.1038/s41598-020-72440-6

**Published:** 2020-10-07

**Authors:** Elisa Stella, Lorenzo Mari, Jacopo Gabrieli, Carlo Barbante, Enrico Bertuzzo

**Affiliations:** 1 Institute of Polar Sciences, Consiglio Nazionale delle Ricerche, Via Torino, 155, 30172 Mestre-Venice, Italy; 2 Dipartimento di Elettronica, Informazione e Bioingegneria, Politecnico di Milano, Via Ponzio 34/5, 20133 Milan, Italy; 3 Department of Environmental Sciences, Informatics and Statistics, Ca’ Foscari University of Venice, Scientific Campus, Via Torino, 155, 30172 Mestre-Venice, Italy

**Keywords:** Climate-change impacts, Ecological epidemiology

## Abstract

A recent outbreak of anthrax disease, severely affecting reindeer herds in Siberia, has been reportedly associated to the presence of infected carcasses or spores released from the active layer over permafrost, which is thawing and thickening at increasing rates, thus underlying the re-emerging nature of this pathogen in the Arctic region because of warming temperatures. Anthrax is a global zoonotic and epizootic disease, with a high case-fatality ratio in infected animals. Its transmission is mediated by environmental contamination through highly resistant spores which can persist in the soil for several decades. Here we develop and analyze a new epidemiological model for anthrax transmission that is specifically tailored to the Arctic environmental conditions. The model describes transmission dynamics including also herding practices (e.g. seasonal grazing) and the role of the active layer over permafrost acting as a long-term storage of spores that could be viable for disease transmission during thawing periods. Model dynamics are investigated through linear stability analysis, Floquet theory for periodically forced systems, and a series of simulations with realistic forcings. Results show how the temporal variability of grazing and active layer thawing may influence the dynamics of anthrax disease and, specifically, favor sustained pathogen transmission. Particularly warm years, favoring deep active layers, are shown to be associated with an increase risk of anthrax outbreaks, and may also foster infections in the following years. Our results enable preliminary insights into measures (e.g. changes in herding practice) that may be adopted to decrease the risk of infection and lay the basis to possibly establish optimal procedures for preventing transmission; furthermore, they elicit the need of further investigations and observation campaigns focused on anthrax dynamics in the Arctic environment.

## Introduction

Recent climate changes have significantly affected human and natural systems. In particular, evidence shows that climate warming has amplified effects on the Arctic, which is experiencing unprecedented changes and challenges from both an environmental and a socio-economic perspective (see e.g. the IPCC Fifth Assessment Report (AR5)^1^).

In recent years particular attention has been devoted to the process of permafrost degradation and to the increasing thawing rates of the active layer. Indeed, not only do these processes produce sizable impacts on landscape degradation, ecological and hydrological systems, infrastructure stability and carbon release^2–6^, which in turn is expected to increase global warming^5,7^, but they may also bring to light at a fast rate contaminants (such as persistent organic pollutants, mercury and others) that were deposited and stored over the years^8,9^. Through similar dynamics, increasing thaw of the active layer also represents a threat because of the potential re-emergence of pathogens, chiefly among which anthrax, which may pose serious risks for local communities and their sustenance^10–13^.

Anthrax generally occurs in nature as a global zoonotic and epizootic disease caused by the spore-forming bacterium *Bacillus anthracis*. It principally affects herbivores and induces high mortality among livestock, entailing significant sanitary and economic consequences, particularly among pastoralist communities. Wild and domestic herbivores usually get infected while grazing, specifically when they ingest spores that have been shed in the soil by the carcasses of dead infected animals. Transmission to humans is less frequent and is caused by contact with either infected animals or their products (e.g. meat, hides, bones and other materials)^14^. The bacterium can turn into a dormant form when it comes into contact with oxygen and atmospheric air. These dormant stages (spores) may spread in the environment and, under favorable conditions, revive again. Spores are remarkably resistant and fairly adaptive to adverse environmental conditions, and can remain viable for decades^14–16^.

Anthrax disease is distributed almost globally, but it principally occurs in sub-Saharan Africa, Asia and America, including also some regions of North America and Canada^14,17,18^. However, recent studies have highlighted the impact of warming temperatures on anthrax suitability in northern latitudes subject to various changes in habitat, wildlife and other environmental variables^18,19^. Indeed, as a consequence of global warming, anthrax has recently occurred at northern latitudes where it was usually unlikely. For this reason, this disease has been identified as a climate-sensitive infection^18,20,21^. A large outbreak of anthrax disease was reported in 2016 in the Yamalo-Nenets region, in the Arctic Russian Siberia, causing the death of one person and over 2000 reindeer. In this case, experts believe that high thawing rates may have led long-buried infected carcasses to surface again, thus resulting in spore release uplift, and ingestion by feeding animals^12,22^, as also stated in the reports edited by the World Organisation for Animal Health (OIE) (World Animal Health Information Database—WAHIS Interface, https://www.oie.int/wahis_2/public/wahid.php/Reviewreport/Review/viewsummary?fupser=&dothis=&reportid=20689). Several studies investigated this episode and pointed out the necessity of enhanced surveillance systems and effective regulation in this territory^23–25^. For instance, the Taimyr peninsula has been estimated to be at high anthrax risk^26^.

Besides the specific problem of the improper burial of carcasses, the dynamics of anthrax spores in frozen soils is thought to be markedly different with respect to other environments. Indeed, when the soil freezes, it acts as a long-term storage of spores. Only during the limited period of the yearly thawing of the active layer, could processes like e.g. cryoturbation, soil cracking and solifluction possibly make anthrax spores viable again for transmission. These insights suggest that the seasonal and inter-annual dynamics of the active layer, and of the top surface of permafrost, can critically control the viability of spores and, in turn, the risk of triggering anthrax outbreaks.

Existing epidemiological models of anthrax transmission follow the formulation originally proposed in 1981 by Hahn and Furniss^27^, who identified two driving mechanisms of pathogen diffusion: environmental contamination and direct contact between susceptible and infected animals. Later, the same authors studied thresholds effects in the case of environmental contamination^28^. Other contributions have then considered additional processes, such as animal migration^29^, vaccination, carcass disposal, and latency in transmission (with a proposed incubation period of about 1–14 days)^14,30,31^. Recently, carcass decay and animal recovery have also been included^32^. Finally, a recent work focused on the interaction between host population dynamics and external environmental factors, which result decisive in defining frequency and amplitude of outbreaks^33^.

However, a mathematical formulation accounting for the peculiarities of the Arctic environment, and in particular for the role of the seasonally thawed layer, is still missing. In this sense, the present study aims to provide a new epidemiological model for anthrax transmission tailored for the Arctic region. Specifically, we propose to model the active layer and the top layer of permafrost, which may be involved in exceptional thawing events, as a long-term ground storage reservoir of anthrax spores, which can resurface during a thaw. In the following sections, we present the transmission model and analyse conditions enabling sustained disease transmission. After studying the model assuming constant parameters, we investigate, by means of Floquet theory^34–37^, the role played by periodic fluctuations, which are particularly important for the problem at hand because both climate and herding practices in the Arctic region are characterized by strong seasonality. Finally, we use real records of active layer thawing depth to discuss its relationship with the risk of anthrax outbreaks.

## Methods

In this section, we first present the general formulation of our anthrax transmission model following both a deterministic and a stochastic approach. The latter seems particularly suitable in this case because of its ability to capture the dynamics of discrete events furthering pathogen spread, especially in the case of a small host population or episodic disease transmission. Then, we illustrate the methods used to derive conditions for the establishment of sustained disease transmission, in particular considering seasonal variations of environmental forcings and herding practice.

### The anthrax transmission model

Our formulation builds on a compartmental model describing the epidemiological dynamics that affect a target population (composed of susceptible and infected individuals) being exposed to environmental contamination. We focus on domestic herbivores because they are both the most at risk and the most socio-economically valuable for Arctic communities. We thus neglect any direct interaction between infected carcasses and carnivores or scavengers, and consider environmental spores as the only source of infection (i.e. by ingestion while herds graze). As little is known yet on how age and sex of the animals may influence anthrax transmission, we suppose that all animals are equally vulnerable to the infection.

Let *S*(*t*), *I*(*t*) and *R*(*t*) be the total abundances of susceptible, infected and temporarily immune animals at time *t*, respectively, and let *H* be the total size of the animal population. Differently from previous formulations, we introduce two different reservoirs of spores. The first (with abundance $B_1 (t)$) accounts for fresh spores that are released after the death of infected hosts and that are immediately available on the soil surface. As these spores infiltrate, are washed away or get buried, they enter the second reservoir (with abundance $B_2 (t)$), which describes long-term storage in the soil. Anthrax transmission can thus be described by the following system of ordinary differential equations:

1 $$\begin{aligned} \frac{dS}{dt}&= \mu (H-S) -F(t)S +\rho R \end{aligned}$$

2 $$\begin{aligned} \frac{dI}{dt}&=\sigma F(t)S -(\mu +\alpha ) I \end{aligned}$$

3 $$\begin{aligned} \frac{dR}{dt}&= (1-\sigma )F(t)S - (\mu +\rho ) R \end{aligned}$$

4 $$\begin{aligned} \frac{dB_1}{dt}&=\theta \alpha \frac{I}{A} -(\delta _1+\chi ) B_1 \end{aligned}$$

5 $$\begin{aligned} \frac{dB_2}{dt}&= \chi B_1 -\delta _2 B_2 \end{aligned}$$

**Figure 1:**
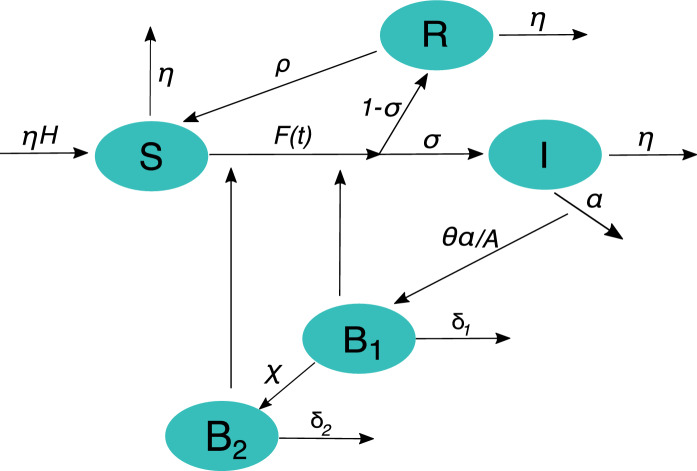
Conceptual diagram of the anthrax transmission model described in Eqs. 1–5.

As for susceptible animals (Eq. 1), we assume that pastoralist practices keep herd size under controlled demographic growth, with $\mu H$ being the constant recruitment rate compensating the natural (non disease-induced) mortality occurring at rate $\mu$. Susceptible animals may become infected at a rate expressed by the total force of infection, *F*(*t*), which will be described in details later. A fraction $\sigma$ of animals that have been exposed to anthrax spores develops symptoms and enters the infected compartment *I* (Eq. 2). Once infected, symptomatic animals may die as a result of the anthrax infection at rate $\alpha$, or die for other causes not related to the disease at rate $\mu$. The remaining fraction of exposed animals ($1-\sigma$, e.g. animals that have been exposed to lower doses of spores) may not exhibit symptom and develop a temporary immunity^38^. As these individuals do not shed spores, we assume that they enter directly the immune compartment *R* (Eq. 3). These animals lose their immunity and return to the susceptible class at rate $\rho$. When infected animals die of anthrax disease, spores proliferate in the host carcass. We assume that each death produces a constant number $\theta$ of spores, which are then released from the carcass and contaminate the surrounding environment, whose areal extent is *A* (Eq. 4). Both $B_1 (t)$ and $B_2 (t)$ are environmental densities of spores per unit area (# spores m$^{-2}$). The freshly released spores decay at a rate $\delta _1$, or may be removed from the surface soil layer and stored in the active layer reservoir at a rate $\chi$ (Eq. 5). The latter rate thus conceptually encapsulates the combined effect of infiltration, runoff, and the burying of infected carcasses without appropriate sanitary precautions. Spores stored in the active layer decay at a rate $\delta _2$. The main processes involved in anthrax transmission dynamics have been conceptualized in Fig. 1.

The force of infection *F*(*t*) , which controls the rate at which susceptible animals get infected, depends on the concentrations of spores ($B_1$, $B_2$) and the rate of exposure ($\beta (t)$) according to

6 $$\begin{aligned} F(t)=\beta (t)\biggl (\frac{B_1}{K+B_1}+\eta (t)\frac{B_2}{K+B_2}\biggl ), \end{aligned}$$

where the fraction $B_i/(K+B_i)$ (for $i=1,2$) is the probability of becoming infected after being exposed to a certain density $B_i$ of spores, *K* being the half-saturation constant (i.e. the dose of spores for which infection risk is half of its maximum value). As mentioned before, because of the processes involving active layer thaw, including e.g. cryoturbation, soil cracking, and solifluction, we assume that spores $B_2$ may become available to grazing animals. The exposure to spores $B_2$ is therefore influenced by active layer thawing, which has a significant seasonal component. The interaction between thawing and the release of spores is expressed through the parameter $\eta (t)$, which quantifies the probability of being exposed to spores $B_2$ relatively to that of being exposed to freshly released spores ($B_1$). Because all the processes mentioned above are more likely to occur with thawing, we assume the probability $\eta (t)$ to be proportional to the depth of the active layer. We will later relax this simple assumption and investigate more complex relationships. We initially mimic the annual cycle of active layer thawing with a simple sinusoidal function (which also simplifies stability analysis via Floquet theory), so that $\eta (t)$ can be expressed as:

7 $$\begin{aligned} \eta (t)=\max \biggl (0, \; \epsilon _{\eta } \sin \biggl (\frac{2\pi }{365}t\biggl )\biggl ) \end{aligned}$$

with $\epsilon _{\eta }$ indicating the maximum amplitude of seasonal fluctuations, i.e. the maximum probability for susceptibles to be exposed to spores $B_2$ relative to spores $B_1$. Note that the soil thaws only during the warmer months, during which susceptibles are potentially exposed to spores $B_2$ ($\eta (t)>0$). Later on, we model more realistically the annual cycle of active layer depth, relating it to a real record of surface soil temperatures via the Stefan equation^39, 40^, so as to better analyze anthrax risk in the Arctic environment.

Herding practices and grazing activity might vary seasonally as well, favouring increased exposure during warmer months. Therefore, we set

8 $$\begin{aligned} \beta (t)= \beta _0\biggl (1+\epsilon _{\beta }\sin \biggl (\frac{2 \pi }{365} t+2\pi \phi \biggl )\biggl ) \end{aligned}$$

where $\beta _0$ is the average value of $\beta (t)$, $\epsilon _{\beta }$ is the maximum amplitude of seasonal grazing fluctuations, while $0\le \phi \le 1$ is the temporal lag between the phases of $\beta (t)$ and $\eta (t)$. Note that *t* is expressed in days.

Finally, to reduce the number of model parameters, we introduce the dimensionless spore concentrations $B^{*}_1={B_1}/{K}$ and $B^{*}_2={B_2}/{K}$ (see equations S1–S5 in the Supplementary Information). This substitution allows the aggregation of parameters $\theta$, *A*, and *K* into a single one, namely $\theta ^{*}=\theta /(A K)$.

### Stochastic formulation

To build a stochastic version of our anthrax transmission model, we rely on an extension of the classic exact stochastic simulator algorithm (SSA)^41^ that has recently been proposed to describe the Haiti cholera epidemic^42^. In the SSA, each animal is considered individually, i.e. the abundances of susceptible and infected animals are treated as discrete variables, ${\mathscr {S}}(t)$ and ${\mathscr {I}}(t)$. Accordingly, each individual experiences stochastic events (i.e. birth, death, infection, anthrax-related death, etc.; see Table 1) that occur at different rates, $e_k$, where *k* indicates a generic event, depending on the state of the system. The overall occurrence of events is modeled as a Poisson point process whose rate *e* is defined as the sum of the rates of occurrence of all possible events, i.e.

$$\begin{aligned} e=\sum \limits _{k=1}^8 e_k . \end{aligned}$$

The inter-arrival time between two subsequent events is thus an exponentially distributed random variable with mean 1/*e*, and the next event to occur is selected according to the probability $e_k/e$^41^.

**Table 1:** State transitions and rates of all possible events involving susceptible, infected and temporally immune (recovered) animals.

Event	State transition	Event rate
Birth of a susceptible	$\mathscr {(S,I,R)}\rightarrow \mathscr {(S}+1\mathscr {,I,R)}$	$e_1=\mu H$
Death of a susceptible	$\mathscr {(S,I,R)}\rightarrow \mathscr {(S}-1\mathscr {,I,R)}$	$e_2=\mu {\mathscr {S}}$
Symptomatic infection	$\mathscr {(S,I,R)}\rightarrow ({\mathscr {S}}-1,{\mathscr {I}}+1,{\mathscr {R}})$	$e_3=\sigma {\mathscr {F}}(t) {\mathscr {S}}$
Death of an infected	$\mathscr {(S,I,R)}\rightarrow \mathscr {(S,I}-1, {\mathscr {R}})$	$e_4=\mu {\mathscr {I}}$
Anthrax-related death	$\mathscr {(S,I,R)}\rightarrow \mathscr {(S,I}-1,{\mathscr {R}})$	$e_5=\alpha {\mathscr {I}}$
Asymptomatic infection	$\mathscr {(S,I,R)}\rightarrow ({\mathscr {S}}-1,{\mathscr {I}},{\mathscr {R}}+1)$	$e_6=(1-\sigma ){\mathscr {F}}(t) {\mathscr {S}}$
Death of a recovered	$\mathscr {(S,I,R)}\rightarrow \mathscr {(S,I,R}-1)$	$e_7=\mu {\mathscr {R}}$
Immunity loss	$\mathscr {(S,I,R)}\rightarrow ({\mathscr {S}}+1,{\mathscr {I}},{\mathscr {R}}-1)$	$e_8=\rho {\mathscr {R}}$

The concentrations of anthrax spores, ${\mathscr {B}}^{*}_1(t)$ and ${\mathscr {B}}^{*}_2(t)$, are instead treated as continuous stochastic variables, because they are typically large enough to allow a continuous description. At each anthrax-related death event, ${\mathscr {B}}^{*}_1(t)$ undergoes a step increase of size $\theta ^{*}$, whereas between events spore concentrations are updated using the analytical solution of equations S4–S5 (see the Supplementary Information), with $\theta ^{*}=0$. In analogy with the deterministic formulation (Eq. 6), the force of infection reads

$$\begin{aligned} {\mathscr {F}}(t)=\beta (t)\biggl (\frac{{\mathscr {B}}^{*}_1}{K+{\mathscr {B}}^{*}_1}+\eta (t)\frac{{\mathscr {B}}^{*}_2}{K+{\mathscr {B}}^{*}_2}\biggl ). \end{aligned}$$

Finally, a Monte Carlo approach, in which many different trajectories (realizations) of the SSA are evaluated, is used to study the long-term behaviour of the stochastic formulation of the anthrax transmission model.

### Derivation of disease transmission conditions

#### Linear stability analysis of time-invariant systems

Conditions for long-term pathogen invasion and sustained transmission (endemicity) are first derived in the absence of seasonal fluctuations. To that end, we consider the exposure rate and the probability to be infected by spores $B_2$ to be constant over time (i.e. $\beta (t)=const=\beta _0$ and $\eta (t)=const=\eta _0$, respectively).

Endemic anthrax transmission is possible if the disease-free equilibrium (DFE), a state of system 1–5 where $(S,I,R,B_1,B_2)=(H,0,0,0,0)$, is asymptotically unstable. Linear stability analysis is used to determine a threshold condition based on the basic reproduction number^43^

9 $$\begin{aligned} R_{0}=\frac{\sigma \beta _0 \theta ^{*} H\alpha (\delta _2+\eta _0\chi )}{\delta _2(\mu + \alpha )(\delta _1+\chi )} . \end{aligned}$$

Specifically, the DFE is asymptotically stable when $R_0<1$ (hence, disease cannot spread in the long run), unstable otherwise (hence, endemic anthrax transmission is possible). The conditions for positivity and asymptotic stability of the endemic equilibrium (EE) of model 1–5 can also be evaluated based on Eq. 9. Specifically, the EE is endowed with strictly positive components and asymptotically stable if $R_0>1$, unfeasible and unstable otherwise. Clearly, $R_0=1$ represents a bifurcation point, where the two equilibria collide and exchange their stability (transcritical bifurcation). For further mathematical details, the reader may refer to the Supplementary Information.

In the absence of the long-term spore reservoir $B_2$, the basic reproduction number ${\tilde{R}}_{0}$ reads:

10 $$\begin{aligned} {\tilde{R}}_{0}=\frac{\sigma \beta _0 \theta ^{*} H \alpha }{(\mu + \alpha )(\delta _1+\chi )}. \end{aligned}$$

This definition will become useful in the following section.

#### Periodic systems: Floquet analysis

Conditions for endemic anthrax transmission to occur in a seasonally forced environment can be studied by applying Floquet theory^36,37^. The disease-free equilibrium of model 1–5 subject to periodic fluctuations is unstable when its maximum Floquet exponent, $\xi _{max}$, is positive (for further theoretical details see Supplementary Information). To compare transmission dynamics between periodic and time-independent conditions we calculated also ${\overline{R}}_0$ by assuming $\eta (t)$ and $\beta (t)$ to be constant and equal to their average value. For any parameter set, ${\overline{R}}_0$ provides information regarding the stability conditions of the system if temporal fluctuations of parameters were neglected.

Note that other parameters may vary seasonally: for instance, the spore transition rate $\chi$ and the decay rates of the spores stored in the two reservoirs may vary over time because of fluctuations in the environment, temperature, and freezing or thawing conditions. However, for the sake of simplicity, and also due to the lack of detailed information, in the following we limit our analyses on the coupled effect of $\beta (t)$ and $\eta (t)$ (Eqs. 8 and 7, respectively).

### Model setting and data

Most of the model parameters have been estimated according to reference values proposed in the literature, as shown in Table 2. The average lifespan of domestic livestock varies widely (on average between 5 and 20 years, or even more^44^), depending on the animal species and herding management. Since animals with shorter life expectancy are more likely to be infected (see Supplementary Fig. S1), we assumed an average mortality rate of 0.2 years$^{-1}$ as a representative case, i.e. domestic cattle with an average lifespan of 5 years. Given high mortality rates among infected herbivores^14^, we assumed that about 70% of infected animals develop symptoms. The remaining 30% grow a temporary immune response, ensuring animal immunity for about 6 months^38^. Typically, infection with anthrax bacterium leads symptomatic animals to death in about 14 days^14^. Then, as the spores are released from infected carcasses, we assumed they remain directly available for about 10 days before their removal from the soil surface^15,32^. Spores can be viable for decades^14^, thus we assumed an average viability of 10 years. Finally, we assumed that the probability $\eta$ can vary between 0 and 1, thus implying that animals can be equally exposed to the two spore reservoirs during the periods of maximum thawing. The parameters $\beta$ and $\theta ^{*}$, which quantify overall exposure and contamination, respectively, are critical in determining transmission dynamics. However, the lack of suitable epidemiological records prevents a proper estimation. Therefore, we explored different combinations of these parameters to compare different scenarios for anthrax transmission dynamics and discuss the results. While the maximum exposure rate $\beta$ has an easily interpretable physical meaning, the parameter $\theta ^{*}$ has a less immediate interpretation. We therefore illustrate results in terms of the corresponding ${\tilde{R}}_{0}$ (Eq. 10), that is, the basic reproduction number of the simplified model that does not account for the long-term spore reservoir $B_2$. All simulations have been run with a total population size of $H=10^4$ animals.

Finally, we exploited real data on the current variability of climate and permafrost dynamics to investigate the relationship between warm years (and related deeper active layers) and the risk of anthrax outbreaks. To that end, we run model simulations using the stochastic formulation and realistic forcing. To obtain the latter, we exploited a 17-year-long (2002–2018) dataset of thawing depth available at the Samoylov monitoring site (Lena River delta, northern Siberia)^45^ which we combined with records of soil surface temperature ($\hbox {T}_{S}$). We then modeled the yearly cycle of the active layer depth *Z* via the Stefan equation^39,40^ according to which $Z=E\sqrt{C_S}$, where *E* is the edaphic factor taking into account soil properties and $C_S$ is the cumulative soil surface temperature, calculated when the top soil temperature is above $0\,^{\circ }\hbox {C}$. The estimated value of parameter *E* is equal to $2.58\,\hbox {cm}^\circ \hbox {C}^{-0.5}$, when *Z* is in cm and $C_S$ in $^{\circ }\hbox {C}$ (see Supplementary Fig. S2). To produce synthetic time series of active layer depth to be used in the simulations, we first calculated the mean annual soil surface temperature and fitted a normal probability distribution to the 17 records. We then produced 200-year-long time-series of daily soil surface temperature randomly sampling the mean annual temperature from the normal distribution and assigning an annual pattern obtained shifting the trajectory of the average year (i.e. the year whose daily values are the averages across the available record for that specific day). Soil surface temperature is then transformed into active layer depth using the calibrated Stefan equation. In each 200-year-long model simulation, we discarded the first 100 years, which were used as model spin-up period, and retained the last 100 years for analysis. We run 100 replicas of the process, thus obtaining a total of 10,000 years of simulated anthrax incidence. Note that a 100-year-long simulation should not be interpreted as a future projection for which the hypothesis of steady climate is hardly justifiable, but rather as a computational way to obtain a large sample of simulations exploring the current climate variability without the need to repeat the spin-up phase of the model.

In the analysis described so far, we assumed the probability of contact between animals and spores $B_2$, i.e. the parameter $\eta (t)$, to be proportional to the active layer depth. This implicitly assumes that the underground concentration of spores is uniform. However, as the potential sources of spores are on the surface, a negative gradient of spore concentration for increasing depth could be expected. Mathematically, this can be mimicked assuming a saturating relationship between the probability $\eta (t)$ and the active layer depth *Z*(*t*) so that the marginal increase of risk associated with a unit increase of *Z* decreases with the depth of the active layer itself. We have therefore explored two scenarios: in the first one (case 1) we assumed a linear relationship, i.e. $\eta (t)\propto Z(t)$; in the second (case 2) a saturating relationship of the type $\eta (t)\propto Z(t)/(Z(t)+Z_0)$, where $Z_0$ represents the semi-saturation depth, which has been set to 0.2 m. We then scaled $\eta (t)$ so that the maximum value for case 1 is equal to 0.2. Accordingly, $\eta (t)$ in case 2 has been scaled so that it has the same long-term mean of case 1.

**Table 2:** Parameter values and their literature sources.

Parameter	Units	Definition	Value	References
$\mu$	[days$^{-1}$]	Baseline mortality rate	$\frac{1}{5\times 365}$	^44^
$\alpha$	[days$^{-1}$]	Disease-related mortality rate	$\frac{1}{14}$	^14^
$\rho$	[days$^{-1}$]	Immunity loss	$\frac{1}{6\times 30}$	^38^
$\delta _1 = \delta _2$	[days$^{-1}$]	Spore decay rate	$\frac{1}{10\times 365}$	^14^
$\chi$	[days$^{-1}$]	Removal rate of freshly released spores	$\frac{1}{10}$	^15, 32^
$\sigma$	[–]	Fraction of symptomatic infected	0.7	^38^
$\eta _0$	[–]	Probability of exposure to thawing-released spores	(0–1)	–
$\epsilon _{\eta }$	[–]	Seasonality of $\eta (t)$	(0–1)	–
$\beta _0$	[days$^{-1}$]	(Average) exposure rate	–	–
$\epsilon _{\beta }$	[–]	Seasonality of $\beta (t)$	(0-1)	–
$\theta$	[spores carcass$^{-1}$]	Environmental spore released from infected carcasses	–	–

The values of the parameters $\eta _0$, $\beta _0$ and $\theta$, for which no references were available, are reported in the Results section, as they may vary for different realizations.

## Results

### Conditions for endemic disease transmission

**Figure 2:**
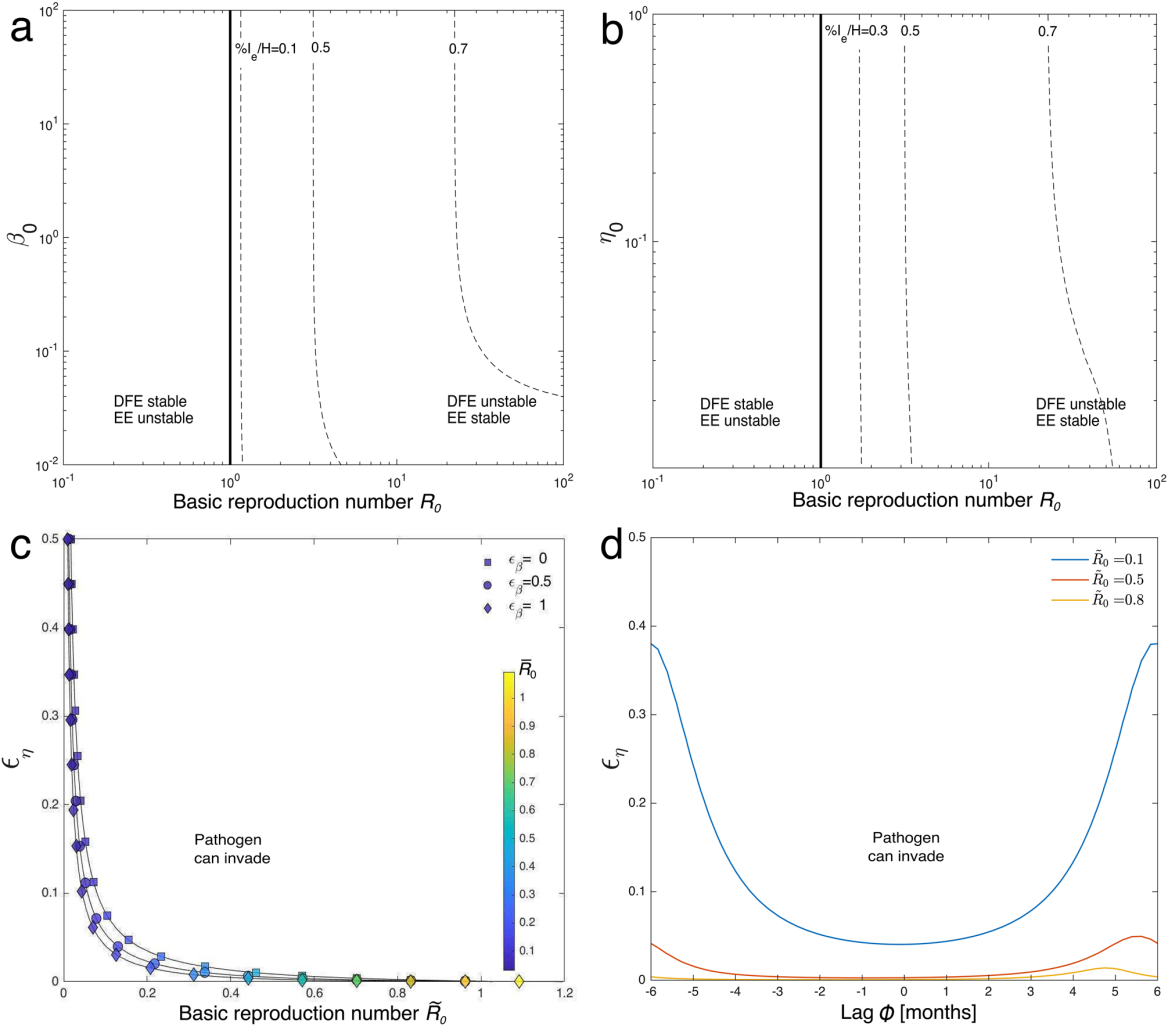
Pathogen invasion conditions with time-invariant dynamics (**a**,**b**) or seasonal variations of the transmission parameters $\eta (t)$ and $\beta (t)$ (**c**,**d**). (**a**,**b**) The DFE and the EE collide and exchange their stability in correspondence of the black thick line (i.e. $R_0=1$). Dashed curves represent the contour levels of the prevalence at the EE, ${\bar{I}}_e/H$, evaluated via numerical simulations of the model 1–5, with $I(0)=10$ and $S(0)=H-I(0)$. (**a**) Prevalence curves for varying exposure rate $\beta _0$ vs $R_0$. (**b**) Prevalence curves for varying probability to thawing-released spores $\eta _0$ vs $R_0$. (**c**) Endemicity thresholds are represented in the ${\tilde{R}}_0$–$\epsilon _{\eta }$ parameter space for different values of seasonality $\epsilon _{\beta }$. The corresponding ${\overline{R}}_0$ is also displayed (coloured markers). (**d**) Endemicity curves in case of lagged signals of $\beta (t)$ and $\eta (t)$ for different values of ${\tilde{R}}_0$. Parameter values as in Table 2. Other parameters: $\beta _0=1$ (**b**,**d**), $\eta _0=0.5$ (a). In panels (**a**,**c**) we set a range of variability for $R_0$ and ${\tilde{R}}_0$ (between 0 and 100) and consequently calculated $\theta$.

The long-term behavior of model 1–5 in the absence of seasonal forcing is shown in panels a and  b of Fig. 2, respectively for different values of $\beta _0$ or $\eta _0$. Endemic transmission is possible to the right of the black thick line, which represents the endemicity condition $R_0 = 1$ (Eq. 9). The prevalence of infection in the population, evaluated from numerical simulations of the deterministic formulation of the model as the fraction of infected animals at the equilibrium (i.e. ${\bar{I}}_e/H$), grows for increasing values of the basic reproduction number. For small $\beta _0$’s (panel a), in particular, high values of anthrax prevalence in the population require large values of $R_0$. On the other hand, for a given value of $R_0$, increments of $\beta _0$ result in an increase of the prevalence of infection, as it would naturally occur if cattle were to be more exposed to the pathogen. However, this pattern is more evident for relatively low values of $\beta _0$, as the prevalence contour line tend to run parallel to the the endemicity threshold for high $\beta _0$’s, as also observed in panel b for varying $\eta _0$’s. In this case, however, the prevalence contour lines tend to run almost parallel to the endemicity curve also for small values of $\eta _0$, except for higher percentages of infected animals, which require higher values of $R_0$ (notice that for $\eta _0=0$, $R_0={\tilde{R}}_0$). The time evolution of the transmission dynamics in the absence of seasonal factors is represented in Fig. S3 in the Supplementary Information.

The effects of seasonally-varying transmission dynamics on long-term pathogen invasion are shown in panels c and d of Fig. 2, respectively for synchronous or lagged fluctuations of the probability of exposure to thaw-released spores, $\eta (t)$ (Eq. 7), and the overall exposure rate, $\beta (t)$ (Eq. 8). In the case of synchronous seasonal variations (panel c), large values of $\epsilon _{\eta }$ and/or $\epsilon _{\beta }$ (the former corresponding to wide fluctuations of $\eta (t)$, which in turn are related to high thawing rates; the latter to ample temporal variations of the overall exposure rate, $\beta (t)$) favor long-term pathogen invasion. In particular, seasonal thaw of the active layer over permafrost makes endemic anthrax transmission possible for values of ${\overline{R}}_0 \ll 1$, particularly for high $\epsilon _{\beta }$. This result has the relevant implication that seasonal fluctuations of exposure and thawing, almost inevitable in the Arctic environment, can sustain anthrax transmission even if the system with constant parameters equal to the corresponding average values could not.

Conversely, asynchronous fluctuations of $\eta (t)$ and $\beta (t)$ (Fig. 2, panel d) may hinder pathogen establishment in the population, thus also possibly slowing or reducing anthrax transmission (for a representative case the reader may refer to Fig. S4 in the Supplementary Information), with the largest effects being observed around a lag of 6 months (i.e. when the two signals are in counterphase).

### Anthrax transmission in a realistic Arctic environment

**Figure 3:**
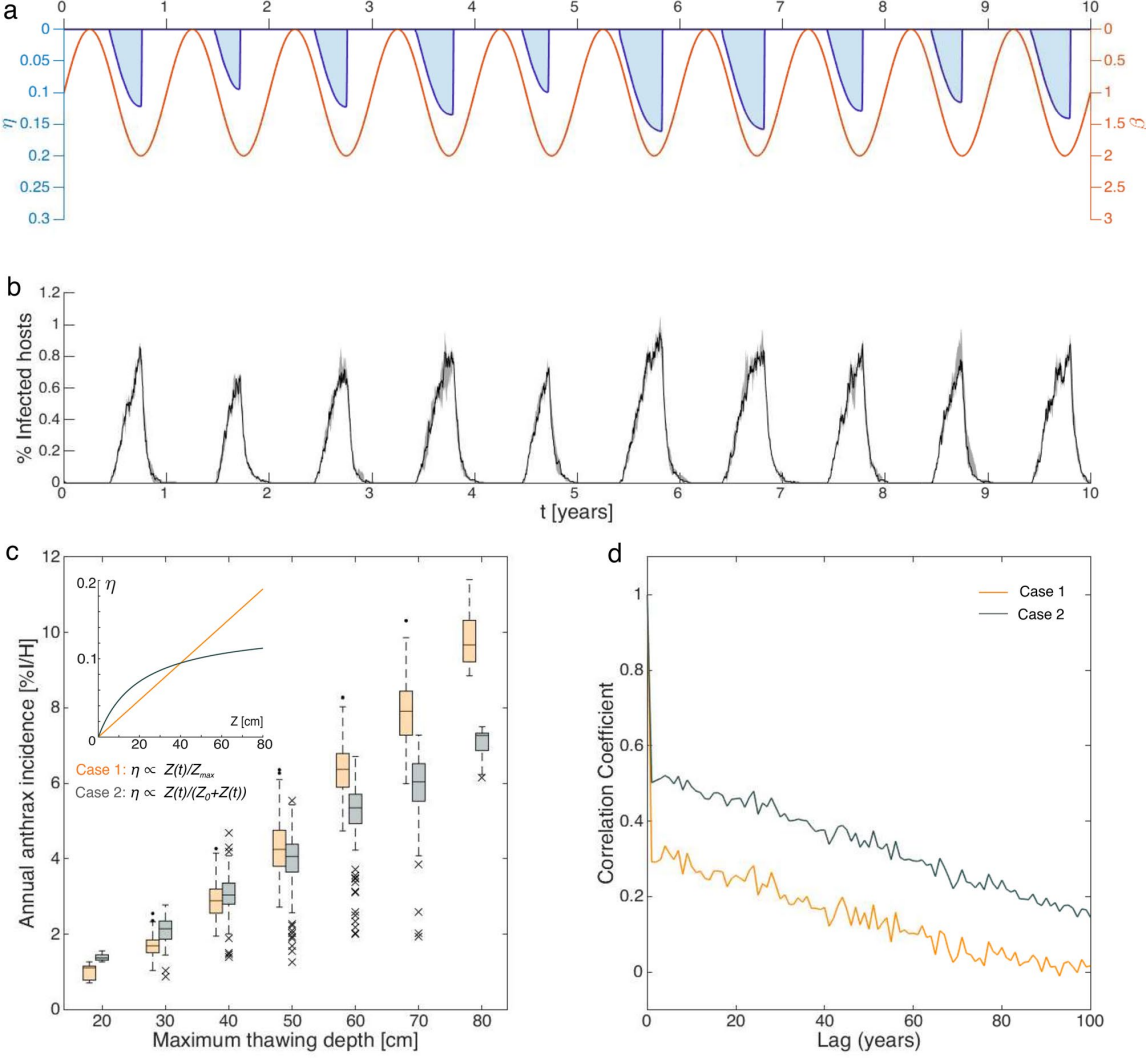
Results from stochastic realizations of the anthrax transmission process forced with time-series of active layer depth derived from the data-set of the Lena River delta monitoring site. (**a**) Model forcing. (**b**) Example of stochastic model simulation. Median (black line) and 5th–95th percentile bounds (gray-shaded area) of 100 model realizations of daily incidence (in percentage over the total population size *H*), over 10 years. (**c**) Box plot of annual cumulative incidence (again, divided by *H*, in percentage) versus maximum thawing depth. Case 1 (in orange) accounts for a linear relation between $\eta$ and *Z*, whereas case 2 (in gray) includes a saturating function, in which $Z_0=20$ cm (inset). In each box, the central mark indicates the median, the bottom and top edges indicate the 25th and 75th percentiles, and the whiskers extend to the 5th and 95th percentiles of the distribution. Outliers are represented as points in case 1 and as “x” marks in case 2. (**d**) Temporal sample autocorrelation of yearly infection risk for case 1 (orange) and case 2 (gray). Initial conditions (**b**,**d**): $I(0)=10$ and $S(0)=H-I(0)$. Parameters values as in Table 2 in the main text. We set ${\overline{R}}_0=2$ and accordingly we derived $\theta ^{*}=2.8\cdot 10^{-6}$. Other parameters: $\beta _0=1$, $\epsilon _{\beta }=1$, $\eta _0=0.2$.

Anthrax transmission at the Lena River site has been simulated starting from the 17-year-long records of active layer depth and soil surface temperature, as described in the Methods section. Model forcings, for a representative period of 10 years, are shown in Fig. 3a, while disease prevalence (abundance of infected hosts in the population), as simulated by 100 realizations of the stochastic formulation of model 1–5, is shown in Fig. 3b. The annual risk of anthrax infection, evaluated as the yearly incidence of disease (total abundance of cases in the population over one year), strongly correlates with the maximum active layer depth in both case 1 and case 2 (Pearson’s $r_1 = 0.92$ and $r_2 = 0.87$ for median risk versus depth, respectively in the first and second case; Fig. 3c). Indeed, deep active layers have a major impact on the risk of anthrax transmission, which proportionally is higher in warmer years, as the probability to be exposed to spores $B_2$ increases. However, a lower annual incidence for large active layer depth is observed in case 2, with a pattern reminiscent of the $\eta$ vs *Z* relationship. It is also interesting to note that, although the average value of $\eta (t)$ is the same, case 2 has an overall lower mean incidence (4.0% vs 4.5%).

Finally, the yearly risk of infection shows a marked (and positive) autocorrelation over a temporal window of at least 10–20 years after which the memory of the system fades away (Fig. 3d). Such behavior is more pronounced in case 2 than in case 1. Therefore, interestingly, the transmission risk at a given point in time is expected to partially affect the magnitude of future anthrax outbreaks, because the spores released by infected carcasses may remain available on the soil for several years.

## Discussion

In this paper, we have proposed and investigated a novel mathematical formulation for anthrax transmission especially tailored for the Arctic environment. Differently from previous formulations^27–33^, in our model we have made explicit the role played by the active layer and the top surface of permafrost in the long-term storage of spores. As a matter of fact, these layers may thaw during particularly warm years, as more frequently occurs due to climate change, thus possibly allowing re-circulation of anthrax spores. Accordingly, we have introduced a formal distinction between spores that are freshly released from infected carcasses versus those that may become viable to infect animals again as a consequence of thawing processes like cryoturbation, solifluction and soil cracking. With the help of the model, we have studied the risk raised by thawing on the re-emergence of anthrax disease in the Arctic, especially insofar it may affect herds of domestic herbivores, which are among both the most vulnerable hosts to this disease and the most important means of sustenance for indigenous communities.

Both a deterministic and a stochastic approach have been applied to study anthrax transmission. With the former, which is particularly amenable to analytical treatment, we have found that the seasonal forcing imposed by thawing and herding practice may favor endemic disease transmission, even under conditions that would prevent it in a host community not subject to seasonal fluctuations. With the latter, which instead allows a discrete, event-based description of infection dynamics in relatively small host populations, and which may thus be particularly appropriate for the problem at hand, we have shown how actual active-layer thaw data can be used to study the risk of anthrax transmission in a realistic Arctic setting.

Our formulation builds on a widely used branch of compartmental epidemiological models, whose simplicity and reliability permit to easily adapt their formulation to a specific objective. However, some assumptions are necessary. In our case, we introduced some simplifying hypotheses on the demographic and epidemiological dynamics of the host population, namely neglecting any distinctions of age or sex and direct animal-to-animal disease transmission. We have also neglected any possible influence exerted by individuals external to the herd, such as wild herbivores, scavengers or other carnivores on demographic and disease transmission dynamics. Yet, we believe that these assumptions do not affect the general purpose of this study. It should also be noted that the occurrence of the transmission patterns presented here requires long-term soil contamination with anthrax spores, and the simultaneous presence of animals and environmental conditions suitable to sustain the transmission of *B. anthracis*. These conditions might not occur in many regions of the Arctic where seasonal thaw alone could thus not trigger outbreaks of anthrax in the absence of the other conditions mentioned above.

Our results may also help the identification of knowledge gaps that should be filled in order to improve our understanding of anthrax transmission in the Arctic environment. In particular, our analysis highlighted how the vertical distribution of spores in permafrost soils could significantly affect transmission dynamics. If spores were concentrated in the first shallow soil layers, transmission risk would likely be controlled by the duration of the active layer thawing. Conversely, if no clear gradients were observed, the risk would be controlled by the active layer depth, whose maximum could occur simultaneously with the maximum animal exposure. Therefore, although spore distribution in soils is expectedly highly heterogeneous and thus difficult to characterize, further monitoring to shed light also on such pattern seems advisable.

Understanding anthrax transmission and its related risk of infection in Arctic regions is crucial given the real concern advanced by a number of studies regarding recent estimates of active-layer and permafrost thawing^12,20,22^. Indeed it has been stated clearly that “the Arctic is warming fast”^5^, actually faster than expected from climate projections^5,6^. Globally, permafrost temperature, in the decade 2007–2016, has increased on average of about $0.3\,^{\circ }\hbox {C}$, with a maximum of about $0.39\,^{\circ }\hbox {C}$ detected in the Arctic^46^. As a result, thicker active-layer depths have been reported in most Arctic monitoring sites and, according to the latest SWIPA (Snow, Water, Ice and Permafrost in the Arctic) report, the southern limit of permafrost appears to have retreated northward by at least 25 km^47^.

Given our results, this overall change of the Arctic environment may have evident implications for anthrax transmission dynamics. A 2011 study conducted by Revich and Podolnaya^22^ warned about the potential hazard associated with the presence of cattle burial sites containing carcasses of animals that had died of anthrax during frequent outbreaks occurred between 1897 and 1925 in Arctic regions, where increasing thawing rates might expose infected carcasses and spores to the surface and therefore favor new possible infections. In particular, that study advanced the necessity of targeted activities around those areas in order to monitor the state of the active layer overlying permafrost. The work by Revich and Podolnaya might be considered a sort of premonition in light of the outbreak occurred later in 2016. All these observations should lead authorities to take in serious consideration precautionary measures to prevent large thawing rates from triggering new anthrax outbreaks and disease endemicity. In this sense, the results presented in our study suggest that, subject to conducive environmental conditions, seasonal thawing may indeed lead to an increased risk of disease outbreaks and sustained transmission in Arctic regions. Likewise, prolonged periods of warming temperatures, which have become more frequent in recent years, may favor the re-emergence of stored spores, which, in addition to freshly released spores, may set breeding ground for future infections, increasing the probability of endemic dynamics if no measures are taken to prevent transmission and environmental contamination. Accordingly, we showed that one measure to decrease the risk of infection could be associated to herding practices: for instance, herders, if possible in accordance to local habits and pasture necessities, could avoid months of maximum thawing by anticipating or postponing seasonal grazing (e.g. by moving animals to seasonal migration routes or transhumance sites earlier or later).

Since anthrax has occurred rarely in Arctic regions, local populations have a low awareness of the effective risks associated with it. Additionally, permafrost degradation combined with drivers associated with human activities (e.g. oil and gas exploitation) are changing the traditional cultural heritage of indigenous pastoralist communities, which might be led to alter their herding routes^48,49^. This change might also contribute to increase the probability to cross hazardous areas. Hence, future development of the model proposed here should include spatially explicit elements, such as the spatial distribution and movement patterns of pastoralist communities, animal migration routes, spore displacement caused by large thawing events and, crucially, a realistic description of the suitability of environmental conditions for the persistence of anthrax spores and overall disease transmission. Such an approach, combined with monitoring activities of active-layer and permafrost state in the most-at-risk areas, along with specific investigations of anthrax infection processes within the Arctic environment^23–26^, might be helpful towards mapping anthrax transmission risk around the Arctic and eventually providing targeted prevention measures.

## Supplementary information

Supplementary Information.

## Data Availability

All data used in this study are publicly available. Active layer depth and soil surface temperature datasets have been retrieved from the study conducted by Boike et al.^45^, available in the PANGAEA platform (https://doi.pangaea.de/10.1594/PANGAEA.891142).
