# Supplementary material for: Permafrost dynamics and the risk of anthrax transmission: a modelling study
Source: Sci Rep. 2020 Oct 7;10:16460. doi: 10.1038/s41598-020-72440-6 (PMC7541526; doi:10.1038/s41598-020-72440-6)
Supplement: Supplementary file 1 — Supplementary Information. [file 41598_2020_72440_MOESM1_ESM.pdf]

Permafrost dynamics and the risk of anthrax transmission: A modelling study

Elisa Stella[1], Lorenzo Mari[2], Jacopo Gabrieli[1], Carlo Barbante[3,1], and Enrico Bertuzzo[3,1*]

[1]Polar Sciences Institute, Consiglio Nazionale delle Ricerche, Via Torino, 155, 30172, Mestre-Venice, Italy
[2]Dipartimento di Elettronica, Informazione e Bioingegneria, Politecnico di Milano, Via Ponzio 34/5, 20133, Milano, Italy
[3]Department of Environmental Sciences, Informatics and Statistics, Ca' Foscari University of Venice, Scientific Campus, Via Torino, 155, 30172, Mestre-Venice, Italy
[*]Address Correspondence to enrico.bertuzzo@unive.it

Supplementary Information

Model rescaling

To reduce the number of model parameters, we introduce the dimensionless spore concentrations $B_1^* = \frac{B_1}{K}$ and $B_2^* = \frac{B_2}{K}$, as well as the aggregated contamination parameter $\theta^* = \frac{\theta}{AK}$. Therefore, the general anthrax transmission model assumes the following formulation:

$$\frac{dS}{dt} = \mu(H-S) - F(t)S + \rho R \tag{S1}$$

$$\frac{dI}{dt} = \sigma F(t)S - (\mu+\alpha)I \tag{S2}$$

$$\frac{dR}{dt} = (1-\sigma)F(t)S + (\mu+\rho)R \tag{S3}$$

$$\frac{dB_1^*}{dt} = \theta^*\alpha I - (\delta_1+\chi)B_1^* \tag{S4}$$

$$\frac{dB_2^*}{dt} = \chi B_1^* - \delta_2 B_2^* \tag{S5}$$

with

$$F(t) = \beta(t)\left(\frac{B_1^*}{1+B_1^*} + \eta(t)\frac{B_2^*}{1+B_2^*}\right).$$

The time evolution of model S1–S5 is shown in Figure S3, in absence of seasonal variables, and in Figure S4 considering periodic thawing and exposure rates. In Figure S4 we represent also the temporal pattern of stored spores, B_2^*.

Linear stability analysis of the time-invariant model

To investigate conditions for long-term pathogen invasion and disease endemicity in an environment devoid of seasonal fluctuations, we perform a classic linear stability analysis of model S1–S5. Therefore, in this section, we consider a constant overall exposure rate $\beta(t) = const = \beta_0$ and a constant probability of exposure to permafrost-released spores $\eta(t) = const = \eta_0$.

Pathogens can invade in the long term, thus establishing endemic transmission, if the DFE/EE of model S1–S5 are asymptotically unstable/stable, respectively. To analyze the stability of each steady state, we linearize system S1–S5 and obtain the Jacobian matrix

$$\mathbf{J} = \begin{bmatrix} -(\mu+F(t)) & 0 & \rho & -\beta_0\frac{1}{(B_1^*+1)^2}S & -\beta_0\frac{\eta_0}{(B_2^*+1)^2}S \\ \sigma F(t) & -(\mu+\alpha) & 0 & \sigma\beta_0\frac{1}{(B_1^*+1)^2}S & \sigma\beta_0\frac{\eta_0}{(B_2^*+1)^2}S \\ (1-\sigma)F(t) & 0 & -(\mu+\rho) & (1-\sigma)\beta_0\frac{1}{(B_1^*+1)^2}S & (1-\sigma)\beta_0\frac{\eta_0}{(B_2^*+1)^2}S \\ 0 & \theta^*\alpha & 0 & -(\delta_1+\chi) & 0 \\ 0 & 0 & 0 & \chi & -\delta_2 \end{bmatrix}.$$

Afterwards, we evaluate the general Jacobian matrix in a neighborhood of each equilibrium point and calculate the associated dominant eigenvalue. The equilibrium is asymptotically stable if the real part of the dominant eigenvalue of $\mathbf{J}$ is negative.

As for the DFE, where $(S, I, R, B_1, B_2) = (H, 0, 0, 0, 0)$, by re-arranging the matrix in order to obtain a block triangular form, which is easier to manage, we have

$$\mathbf{J_0} = \begin{bmatrix} -\mu & \rho & 0 & -\beta_0 H & -\beta_0 \eta_0 H \\ 0 & -(\mu+\rho) & 0 & (1-\sigma)\beta_0 H & (1-\sigma)\beta_0 \eta_0 H \\ 0 & 0 & -(\mu+\alpha) & \sigma\beta_0 H & \sigma\beta_0\eta_0 H \\ 0 & 0 & \theta^*\alpha & -(\delta_1+\chi) & 0 \\ 0 & 0 & 0 & \chi & -\delta_2 \end{bmatrix}.$$

The eigenvalues of $\mathbf{J_0}$ are obtained by solving $\det(\lambda I - \mathbf{J_0}) = 0$. However, the block-triangular structure of $\mathbf{J_0}$ makes it immediate to conclude that $-\mu$ and $-(\mu+\rho)$ are two eigenvalues, while the others are the solutions of the characteristic polynomial

$$\lambda^3 + \lambda^2(\mu+\alpha+\chi+\delta_1+\delta_2) + \lambda((\mu+\alpha)(\delta_1+\chi) + \delta_2(\delta_1+\chi+\mu+\alpha) - \sigma\beta_0\theta^*\alpha H) + \\ + \delta_2(\mu+\alpha)(\delta_1+\chi) - \sigma\beta_0\theta^*\alpha H(\delta_2+\eta_0\chi) = 0.$$

By the Routh-Hurwitz stability criterion, the conditions for the stability of DFE are

$$\begin{cases} (\delta_1+\delta_2+\chi+\mu+\alpha)((\mu+\alpha)(\delta_1+\chi)+\delta_2(\delta_1+\chi+\mu+\alpha)-\sigma\beta_0 H\theta^*\alpha) + \\ -\delta_2(\mu+\alpha)(\delta_1+\chi) - \sigma\beta_0\theta^*\alpha H(\delta_2+\eta_0\chi) > 0 \\ \delta_2(\mu+\alpha)(\delta_1+\chi) - \sigma\beta_0\theta^*\alpha H(\delta_2+\eta_0\chi) > 0. \end{cases} \tag{S6}$$

By algebraic manipulation, it is possible to prove that the two inequalities above coincide. They can be written as $R_0 < 1$, where

$$R_0 = \frac{\sigma\beta_0\theta^* H\alpha(\delta_2+\eta_0\chi)}{\delta_2(\mu+\alpha)(\delta_1+\chi)} \tag{S7}$$

is defined as the basic reproduction number. Clearly, the DFE is asymptotically unstable, thus allowing long-term pathogen invasion, if $R_0 > 1$.

As for the EE, first we need to work out an analytical expression for its components $(S^e, I^e, B_1^{*,e}, B_2^{*,e})$. This can be easily accomplished by setting the right-hand sides of model S1–S5 equal to zero, thus obtaining

$$\begin{aligned} B_1^{*,e} &= \frac{\theta^*\alpha}{\delta_1+\chi} I^e \\ B_2^{*,e} &= \frac{\chi}{\delta_2} B_1^{*,e} \\ S^e &= H - \frac{(\mu+\alpha)(\sigma\rho+\mu)}{\mu\sigma(\mu+\rho)} I^e \\ R^e &= \frac{(1-\sigma)(\mu+\alpha)}{\sigma(\mu+\rho)} I^e \\ f(I^e) &= \frac{\beta_0\sigma}{(\mu+\alpha)} \left(\frac{\gamma}{1+\gamma I^e} + \eta_0 \frac{\zeta\gamma}{1+\zeta\gamma I^e} \right) \left(H - \frac{(\mu+\alpha)(\sigma\rho+\mu)}{\mu\sigma(\mu+\rho)} I^e \right) - 1, \end{aligned} \tag{S8}$$

where $\gamma = \frac{\theta^*\alpha}{\delta_1+\chi}$, $\zeta = \frac{\chi}{\delta_2}$ and $f(I^e)$ is a nonlinear function of I^e. We note that $f(I^e)$ is monotonically decreasing for $I^e \geq 0$ and that it tends to a negative value for $I^e \to +\infty$. Furthermore, $f(0) = R_0 - 1 > 0$ for $R_0 > 1$. Hence, there exists a unique positive solution to $f(I^e) = 0$, corresponding to the infected component of the EE, whose value can be easily computed numerically. Numerically, it is also straightforward to verify that if $R_0 > 1$ the EE is not only feasible (i.e. characterized by strictly positive components), but also asymptotically stable. Therefore, at $R_0 = 1$ a transcritical bifurcation occurs, at which the DFE and the EE collide and exchange stability: if $R_0 < 1$, the DFE is stable, while the EE is unfeasible (and unstable); if $R_0 > 1$, the DFE is unstable, while the EE is feasible and stable.

Endemicity conditions under seasonal forcing

In the presence of seasonal (periodic) fluctuations of the model parameters, the stability properties of the DFE of model S1–S5 are determined by the Floquet exponents associated with the time-periodic Jacobian matrix

$$\mathbf{J_0(t)} = \begin{bmatrix} -\mu & \rho & 0 & -\beta(t)H & -\beta(t)\eta(t)H \\ 0 & -(\mu+\rho) & 0 & (1-\sigma)\beta(t)H & (1-\sigma)\beta(t)\eta(t)H \\ 0 & 0 & -(\mu+\alpha) & \sigma\beta(t)H & \sigma\beta(t)\eta(t)H \\ 0 & 0 & \theta^*\alpha & -(\delta_1+\chi) & 0 \\ 0 & 0 & 0 & \chi & -\delta_2 \end{bmatrix}.$$

where

$$\beta(t) = \beta_0\left(1+\varepsilon_\beta sin\left(\frac{2\pi}{365}t+2\pi\phi\right)\right) \quad \text{and}$$
$$\eta(t) = \max\left(0,\ \varepsilon_\eta \sin\left(\frac{2\pi}{365}t\right)\right)$$

Specifically, the DFE is unstable if and only if the maximum Floquet exponent is larger than zero.

Given the block-triangular form of $\mathbf{J_0}(t)$, we can limit our attention to the subsystem that includes only the infection-related compartments of the model, namely I, B_1^* and B_2^*. Close to the DFE, the dynamics of this infection subsystem are described by the reduced-order Jacobian

$$\mathbf{J_0^*}(t) = \begin{bmatrix} -(\mu+\alpha) & \sigma\beta(t)H & \sigma\beta(t)\eta(t)H \\ \theta^*\alpha & -(\delta_1+\chi) & 0 \\ 0 & \chi & -\delta_2 \end{bmatrix}.$$

Floquet exponents can be numerically calculated by integrating the matrix differential equations[1]

$$\frac{d\mathbf{A}(t)}{dt} = \mathbf{J_0^*}(t)\mathbf{A}(t)$$

over one period (i.e. from $t = 0$ to $t = T = 1$ year) with the identity matrix as initial condition for $\mathbf{A}(0)$. The dominant eigenvalue of the monodromy matrix $\mathbf{A}(T) = \mathbf{A}(1)$ represents the maximum Floquet multiplier, ρ_{max}, from which the maximum Floquet exponent, ξ_{max}, is readily evaluated as

$$\xi_{max} = \frac{ln(\rho_{max})}{T}.$$

Therefore, the DFE of model S1–S5 subject to periodic forcing is unstable if $\xi_{max} > 0$. Numerical codes for the evaluation of the maximum Floquet exponents of seasonally forced systems are available in the literature in MATLAB®[2] and *Mathematica*®[1] languages.

1 Supplementary figures

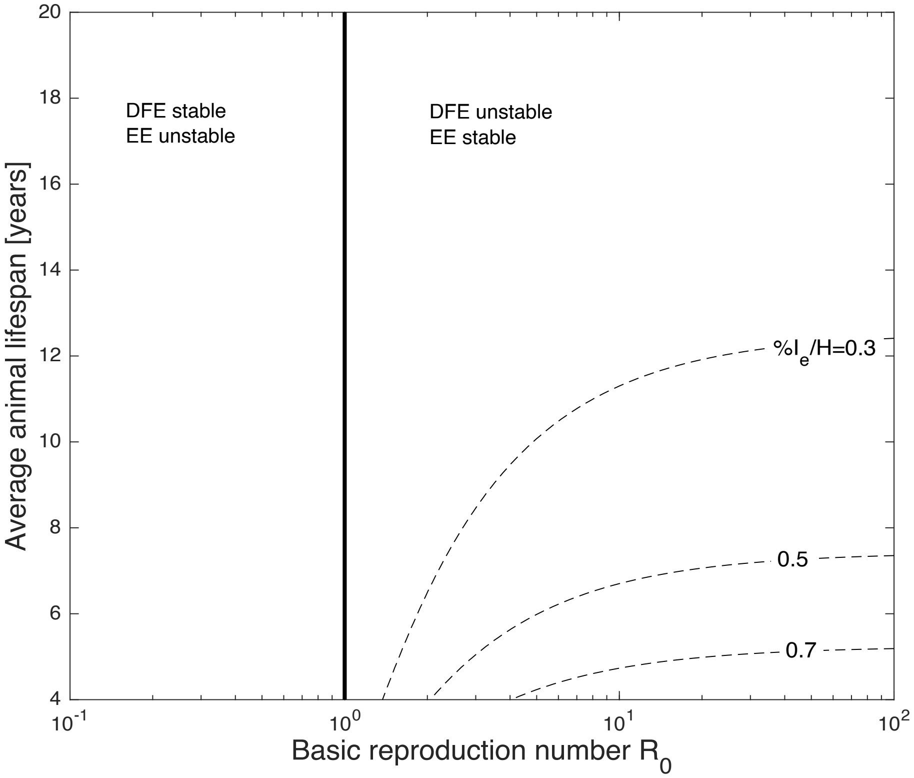

Figure S1. Pathogen invasion conditions with time-invariant dynamics. The DFE and the EE collide and exchange their stability in correspondence of the black thick line (i.e. $R_0 = 1$). Dashed curves represent the contour levels of $\bar{I}_e/H$, evaluated numerically (by setting $I(0) = 10$ and $S(0) = H - I(0)$) for different values of μ, so that lifespan $1/\mu$ varies between 4 and 20 years. Parameter values as in Table 2 in the main text (except for μ). Other parameters: $\beta_0 = 1$, $\eta_0 = 0.5$.

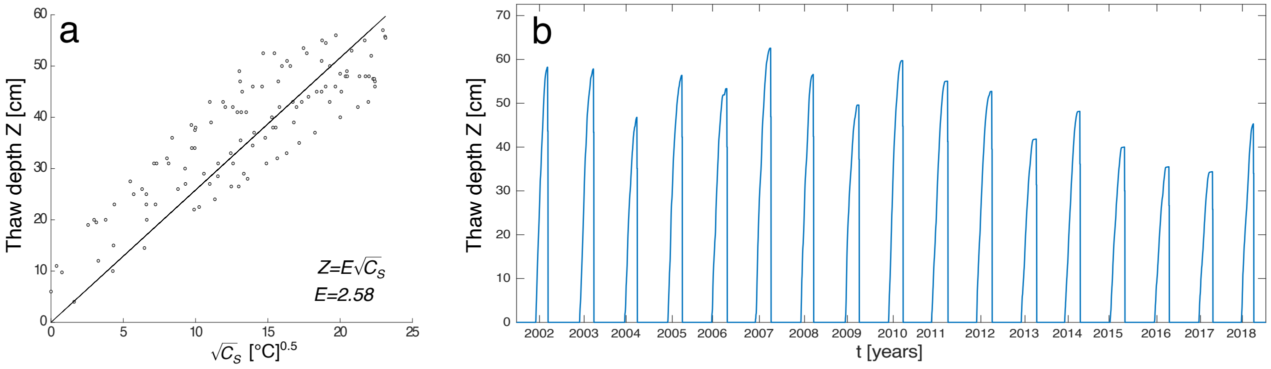

Figure S2. Derivation of daily time-series of active layer thawing from the Stefan equation. (a) Calculation of the edaphic factor by comparing the square root of the cumulative soil temperature $\sqrt{C_S}$ vs. thaw depth Z, according to the Stefan equation showed in the plot . (b) 17-year-long time-series of active layer measurements at the Lena River Site (Northern Siberia). While the thawing phase is derived according to Stefan formula, the freezing process has been described as a rapid quadratic trend evaluated in the three days after the end of thawing (i.e. when surface temperature falls below 0°).

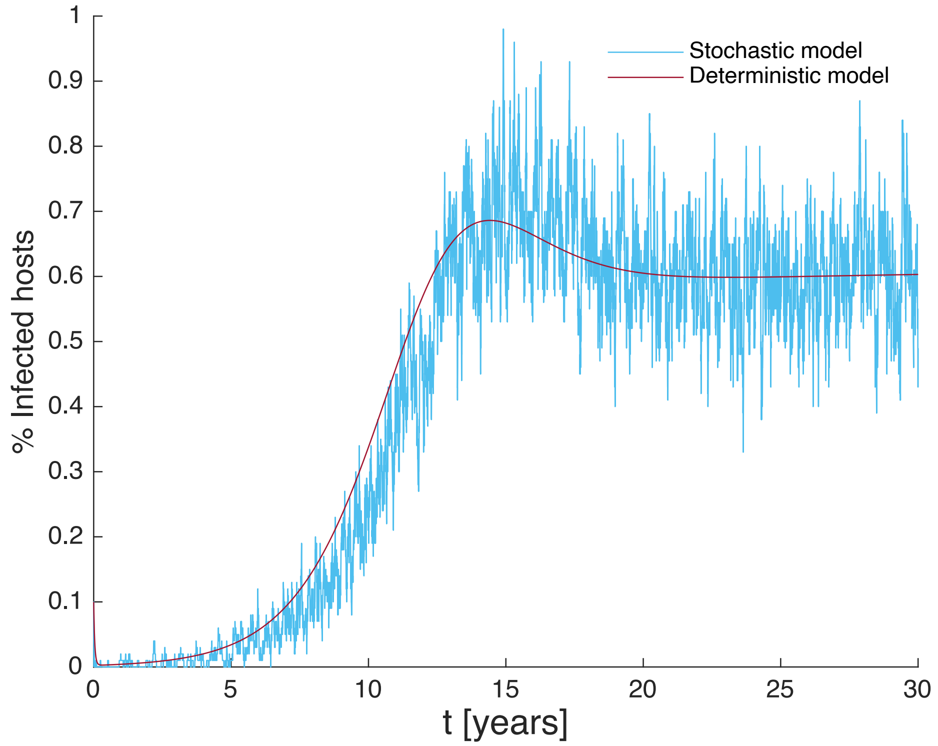

Figure S3. Simulation of the anthrax transmission model. Time-invariant case, where seasonal fluctuations of the transmission parameters are not accounted for. Initial conditions: $I(0) = 10$ and $S(0) = H - I(0)$. Parameters values as in Table 2 in the main text. We set $R_0 = 2$ and accordingly we derived $\theta^* = 2.8 \cdot 10^{-6}$. Other parameters: $\beta_0 = 1$, $\varepsilon_\beta = 1$, $\eta_0 = 0.2$.

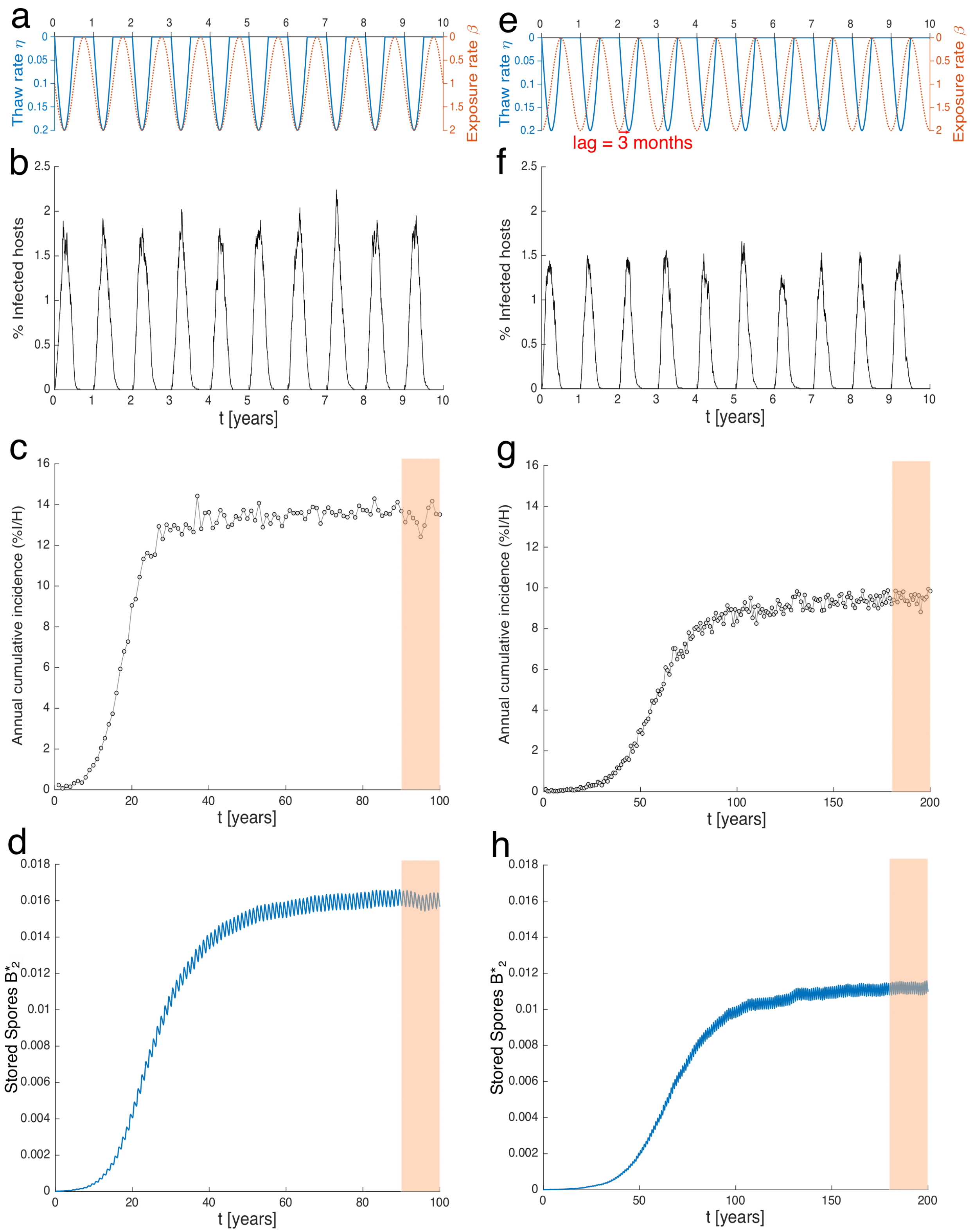

Figure S4. Simulation of the anthrax transmission model with seasonal forcings $\eta(t)$ and $\beta(t)$ (panels a and e). Panels (a–d) represent synchronous signals, while in panels (e–h) peaks of $\beta(t)$ occur with a lag of 3 months after those of $\eta(t)$. (b),(f) percentage of infected hosts ($\%I/H$), evaluated after the disease has reached the endemic status. (c),(g) Annual incidence (i.e. number of animals infected in % over H). (d),(h) Number of spores stored in the active layer B_2^*. The light red box indicates the 10-years window showed in panels (a),(d). Initial conditions: $I(0) = 10$ and $S(0) = H - I(0)$. Parameters values as in Table 2 in the main text. We set $R_0 = 2$ and accordingly we derived $\theta^* = 1.2 \cdot 10^{-6}$. Other parameters: $\beta_0 = 1$, $\varepsilon_\beta = 1$, $\eta_0 = 0.2$.

References

1. Klausmeier, C. A. Floquet theory: A useful tool for understanding nonequilibrium dynamics. *Theor. Ecol.* **1**, 153–161, DOI: 10.1007/s12080-008-0016-2 (2008).
2. Mari, L., Casagrandi, R., Bertuzzo, E., Rinaldo, A. & Gatto, M. Floquet theory for seasonal environmental forcing of spatially explicit waterborne epidemics. *Theor. Ecol.* **7**, 351–365, DOI: 10.1007/s12080-014-0223-y (2014).
